# Comprehensive structural variation genome map of individuals carrying complex chromosomal rearrangements

**DOI:** 10.1371/journal.pgen.1007858

**Published:** 2019-02-08

**Authors:** Jesper Eisfeldt, Maria Pettersson, Francesco Vezzi, Josephine Wincent, Max Käller, Joel Gruselius, Daniel Nilsson, Elisabeth Syk Lundberg, Claudia M. B. Carvalho, Anna Lindstrand

**Affiliations:** 1 Department of Molecular Medicine and Surgery, Karolinska Institutet, Stockholm, Sweden; 2 Science for Life Laboratory, Karolinska Institutet Science Park, Solna, Sweden; 3 Center for Molecular Medicine, Karolinska Institutet, Stockholm, Sweden; 4 Science for Life Laboratory, Department of Biochemistry and Biophysics, Stockholm University, Stockholm, Sweden; 5 Department of Clinical Genetics, Karolinska University Hospital, Stockholm, Sweden; 6 Science for Life Laboratory, School of Engineering Sciences in Chemistry, Biotechnology and Health, KTH Royal Institute of Technology, Stockholm, Sweden; 7 Science for Life Laboratory, Department of Biosciences and Nutrition, Karolinska Institutet, Stockholm, Sweden; 8 Department of Molecular and Human Genetics, Baylor College of Medicine, Houston TX, United States of America; University of Pennsylvania, UNITED STATES

## Abstract

Complex chromosomal rearrangements (CCRs) are rearrangements involving more than two chromosomes or more than two breakpoints. Whole genome sequencing (WGS) allows for outstanding high resolution characterization on the nucleotide level in unique sequences of such rearrangements, but problems remain for mapping breakpoints in repetitive regions of the genome, which are known to be prone to rearrangements. Hence, multiple complementary WGS experiments are sometimes needed to solve the structures of CCRs. We have studied three individuals with CCRs: Case 1 and Case 2 presented with *de novo* karyotypically balanced, complex interchromosomal rearrangements (46,XX,t(2;8;15)(q35;q24.1;q22) and 46,XY,t(1;10;5)(q32;p12;q31)), and Case 3 presented with a *de novo*, extremely complex intrachromosomal rearrangement on chromosome 1. Molecular cytogenetic investigation revealed cryptic deletions in the breakpoints of chromosome 2 and 8 in Case 1, and on chromosome 10 in Case 2, explaining their clinical symptoms. In Case 3, 26 breakpoints were identified using WGS, disrupting five known disease genes. All rearrangements were subsequently analyzed using optical maps, linked-read WGS, and short-read WGS. In conclusion, we present a case series of three unique *de novo* CCRs where we by combining the results from the different technologies fully solved the structure of each rearrangement. The power in combining short-read WGS with long-molecule sequencing or optical mapping in these unique *de novo* CCRs in a clinical setting is demonstrated.

## Author summary

Unexpected complexities are common findings in the breakpoints of karyotypically balanced complex chromosomal rearrangements (CCRs). Such findings are of clinical importance, as they may be the cause of mendelian phenotypes in the rearrangement carrier. Whole genome sequencing (WGS) allows for high resolution characterization of CCRs, but problems remain for mapping breakpoints located in repetitive regions of the genome, which are known to be prone to rearrangements. In our study, we use multiple complementary WGS experiments to solve the structures of three CCRs originally identified by karyotyping. In all cases, the genomic structure of the derivative chromosomes was resolved and a molecular genetic explanation of the clinical symptoms of the patients was obtained. Furthermore, we compare the performance, sensitivity and resolution of four different WGS techniques for solving these CCRs in a clinical diagnostic laboratory set.

## Introduction

Complex chromosomal rearrangements (CCRs) are rearrangements involving more than two chromosomes or more than two breakpoints [1, 2]. Balanced, *de novo*, CCRs are extremely rare and more than half are associated with an affected phenotype [3]. Traditionally in a diagnostic laboratory, karyotypically detected complex rearrangements are further analyzed using methods such as array comparative genomic hybridization (aCGH) and fluorescence *in situ* hybridization (FISH). Which method to choose and the number of experiments needed to resolve its genomic structure will be dependent on the characteristics of the rearrangements under scrutiny. These characteristics include the number of potential breakpoints, as well as if the rearrangement includes unbalanced events such as cryptic deletions, which are commonly the cause of the affected phenotype [4, 5]. Additionally, factors such as cost, patient phenotype, time, availability of tests and interests of the lab (diagnostic versus research) will all be considered in deciding which methodology to choose.

More recently, whole genome sequencing (WGS) has been used to study complex rearrangements. Compared to the standard cytogenetic techniques, WGS offers a wide range of advantages, including high resolution and the ability to detect rearrangements of any size and type in a single experiment, providing important clues for the mechanism of formation [6, 7]. Furthermore, it may be critical for diagnosis of genetic diseases as well as for genotype-phenotype studies. However, solving the structure of a complex rearrangement is costly and time consuming, mainly due to the sheer amount of breakpoints involved, the large variety in sizes and types of structural variants involved, challenges in interpretation, as well as specific limitation detection of the WGS method of choice.

Today, Illumina sequencing technology is the most commonly used second-generation WGS technology [8]. Illumina sequencing is cost efficient and provides high quality data with a wide range of library preparation methods available. Each of these library preparation methods produces data at different cost and with different properties. In particular, the long insert-size mate-pair (MP) sequencing protocols have been useful for analyzing structural variation, mainly due to its high span coverage which potentially could bridge regions that are hard to map, such as repetitive regions [7, 9]. On the other hand, the higher coverage PCR-free paired-end (PE) libraries enables the use of split reads for exact breakpoint analysis, and highly precise read depth CNV detection [10, 11]. These different protocols provide a flexible framework for analyzing different types of variations and answering different questions.

However, it is not obvious which method to choose when faced with the problem of solving a complex rearrangement. Even though WGS comes with a range of advantages it has important limitations, including low sensitivity in repetitive regions. Furthermore, haplotyping of long genomic segments is still a challenge for WGS even using single-molecule sequencing technologies. Therefore, phasing multiple rearrangement breakpoints that may have been created concomitantly in complex rearrangements including CCRs and chromothripsis events, can be a daunting task. Moreover, the breakpoints found using WGS commonly needs to be validated using orthogonal techniques, most commonly breakpoint PCR and Sanger sequencing. Hence, even when using WGS, the analysis of complex rearrangements is difficult, and a large number of experiments may be needed.

Bionano optical mapping is a technology that enables detection of large structural variants across the entire genome, potentially helpful to resolve long haplotypes including detection of genomic variants in *cis*. In contrast to the short-read WGS methods commonly used today, Bionano optical mapping utilize long DNA molecules (>100 kb). The usage of long input DNA molecules enables the optical maps to span repetitive and poorly mapped regions of the genome. Further, each optical map may span multiple adjacent breakpoints, providing additional information on how the breakpoints relate to each other. Bionano optical maps show great promise for SV detection and phasing [12], although optical maps are currently limited by lower resolution compared to sequencing technologies and the availability of the technology itself. Linked-read sequencing is a method provided by 10X Genomics, where linked reads are used to detect structural variation and allows for detection of SVs located within repetitive regions using barcoding of long DNA molecules [13, 14] but it is also limited by availability of a comprehensive software and high false-positive rate.

In this study, we have characterized three unique CCRs combining multiple technologies including standard cytogenetic techniques (aCGH and FISH), followed by short- and long-read approaches in addition to Bionano optical mapping. For short-reads, we used two different sequencing protocols, Illumina 30X PCR-free PE WGS and Illumina Nextera MP WGS, whereas linked-read sequencing using 10X Genomics Chromium was used to obtain synthetic long-reads. By combining next generation sequencing and cytogenetic techniques, we were able to fully characterize the molecular structures of each unique CCR. Lastly, we performed a comprehensive comparison of each methodology applied here concerning the resolution and sensitivity to detect SVs of different sizes throughout the genome, which we discuss in detail.

## Results

Results for the cytogenetic studies are presented in S1 Appendix. BAM files containing all supporting reads for the three rearrangements are available at European Nucleotide Archive (ENA), project number PRJEB26322, and Bionano xmap files are available in the S1 Dataset.

### Case 1

**Fig 1:**
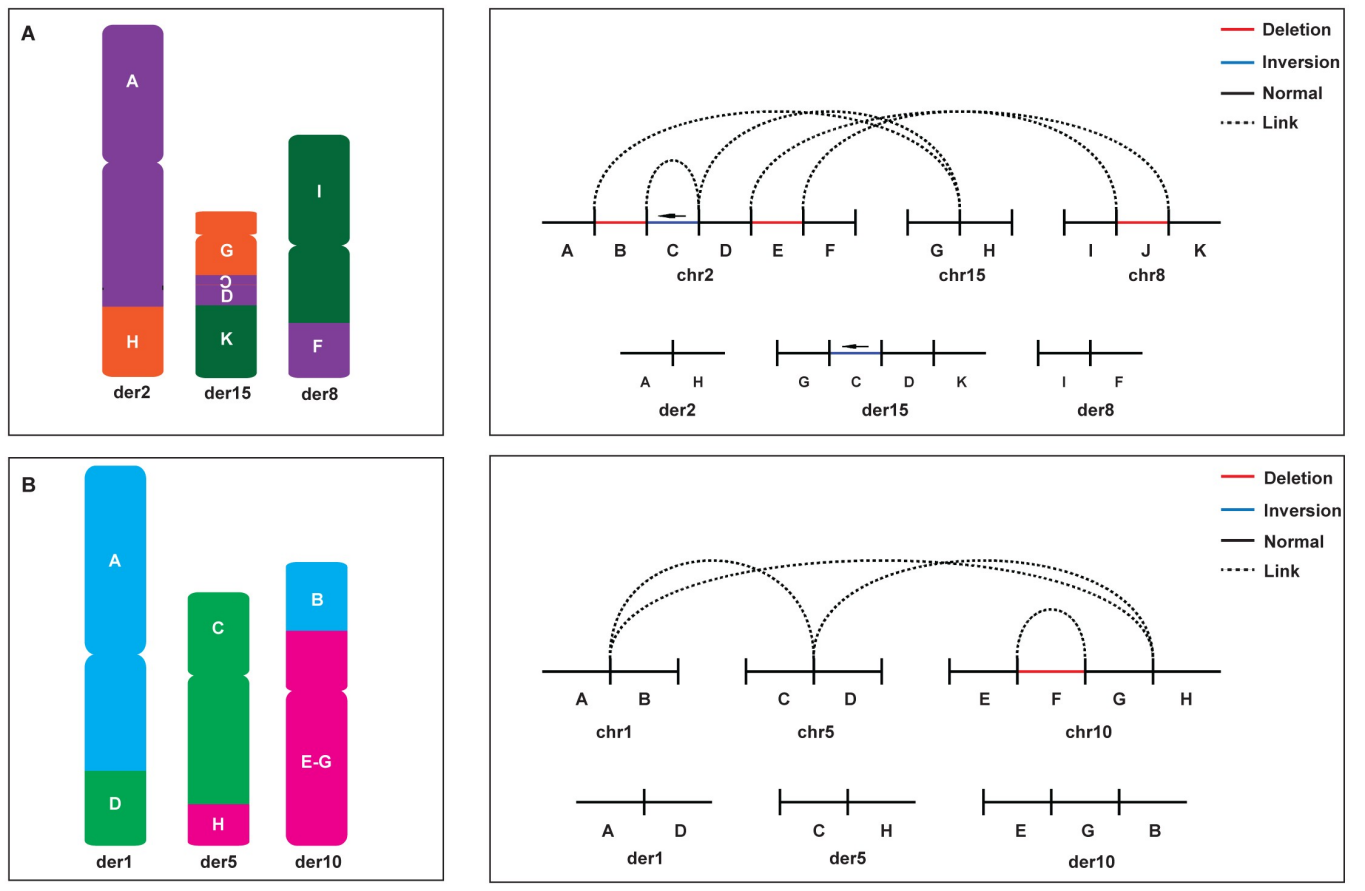
Molecular characterization of two complex interchromosomal rearrangements. **(A**) The complex chromosomal rearrangement identified in Case 1, t(2;8;15)(q34;q23.3;q21.3). A schematic illustration of the derivative chromosomes 2, 8 and 15 is shown to the left. The 11 genomic fragments are labeled from A-K and colored according to the parental chromosomal origin with chromosome 2 in purple, chromosome 8 in green and chromosome 15 in orange. Three fragments from chromosome 2 have been translocated to both derivative 8 and derivative 15 with one fragment inverted (Fragment C, indicated by the orientation of the letter inside the box). To the right, two linear representations of the whole genome sequencing results are shown. On top, the fragments are outlined on the parental chromosomes with links between fragments illustrated as dashed lines. The bottom diagrams show the final derivative chromosomes. For both fragment copy number status is indicated as black (normal) or red (deleted) and inverted orientation is highlighted in blue and marked by an arrow. (**B**) The complex chromosomal rearrangement identified in Case 2, 46,XY,t(1;10;5)(q31.3;p12.31;q23.2), as in A. The eight genomic fragments are labeled from A-H and chromosome 1 origin is shown in blue, chromosome 5 in green and chromosome 10 in pink.

The t(2;8;15) complex rearrangement was first indicated by regular karyotyping, however, this analysis only showed an abnormal derivative chromosome 15 and the karyotype was reported out as 46,XX,del(15)(q?22) (S1 Fig). Further delineating of the chromosomes using FISH and aCGH identified the complex rearrangement involving three chromosomes. Deletions in the breakpoints of chromosomes 2 and 8 were detected using FISH and genome-wide aCGH. Of note, although the 14.5 Mb deletion at 8q23 is within the resolution of classical chromosome analysis, it was not detected, likely masked by the presence of multiple segments involved in the complex rearrangement (S1 Fig). WGS confirmed the aforementioned chromosomal rearrangements and determined the chromosome 2 deletion sizes to be 2.1 Mb and 2.3 Mb, respectively. Additionally, a genomic segment of approximately 970 kb, originally located between the two deleted parts from chromosome 2, was inserted onto chromosome 15 (fragment D, Fig 1A) together with a small inverted 12 kb fragment also originating from chromosome 2 (fragment C, Fig 1A). The first deletion of 2.1 Mb removed eight protein-coding genes and the second deletion of 2.3 Mb deletion removed three protein-coding genes (Table 1). The deletion on chromosome 8 was determined to be 14.5 Mb and removed 49 protein-coding genes (Table 1). The translocation breakpoint on chromosome 15 was balanced with only loss of three nucleotides (Fig 1A, S2 Fig). Analyzing the breakpoint junctions on the nucleotide level, it was observed that no microhomology was present in any of the breakpoint junctions (Table 2, S2 Fig). In one breakpoint junction, there was a four-nucleotide indel at the junction, and a three-nucleotide indel 14 nucleotides upstream, none of them present in the dbSNP database, therefore potentially originated concomitantly to the CCR (S2 Fig). According to the WGS results, the molecular karyotype is t(2;8;15)(q34;q23.3;q21.3) seq[GRCh37] g.[chr2:pter_cen_209425831::chr15:55083064_qter] g.[chr8:pter_cen_114508085::chr2:214880375_qter] g.[chr15:pter_cen_55083061::chr2: 211567929_211580844inv::chr2:211580785_212551796::chr8:129040005_qter]. No other rare structural events were detected in the microarray or WGS data.

**Table 1:** Deletions identified at the breakpoints of the complex chromosomal aberrations.

Case	Karyotype	Hg19 deletion coordinates	Size (Mb)	Genes
1	t(2;8;15)(q34;q23.3;q21.3)	chr2:209,425,211–211,567,929	2.1	*MAP2*, *UNC80*, *RPE*, *KANSL1L*, *ACADL*, *MYL1*, *LANCL1*, *CPS1*
		chr2:212,551,755–214,880,375	2.3	*ERBB4*, *IKZF2*, *SPAG15*
		chr8:114,508,086–129,040,004	14.5	***TRPS1***, *EIF3H*, *UTP23*, *RAD21*, *AARD*, *SLC30A8*, *MED30*, ***EXT1***, *SAMD12*, *TNFRSF11B*, *COLEC10*, *MAL2*, *ENPP2*, *TAF2*, *DSCC1*, *DEPTOR*, *COL14A1*, *MRPL13*, *MTBP*, *SNTB1*, *HAS2*, *ZHX2*, *DERL1*, *TBC1D31*, *FAM83A*, *C8orf76*, *ZHX1*, *ATAD2*, *WDYHV1*, *FBXO32*, *KLHL38*, *ANXA13*, *FAM91A1*, *FER1L6*, *TMEM65*, *TRMT12*, *RNF139*, *TATDN1*, *NDUFB9*, *MTSS1*, *ZNF572*, *SQLE*, *WASHC5*, *NSMCE2*, *TRIB1*, *FAM84B*, *POU5F1B*, *MYC*, *TMEM75*
2	t(1;10;5)(q31.3;p12.31;q23.2)	chr10:4,689,760–19,120,882	14.4	*AKR1E2*, *AKR1C1*, *AKR1C2*, *AKR1C3*, *AKR1C4*, *UCN3*, *TUBAL3*, *NET1*, *CALML5*, *CALML3*, *ASB13*, *FAM208B*, *GDI2*, *ANKRD16*, *FBXO18*, *IL15RA*, *IL2RA*, *RBM17*, *PFKFB3*, *PRKCQ*, *SFMBT2*, *ITIH5*, *ITIH2*, *KIN*, *ATP5C1*, *TAF3*, ***GATA3***, *CELF2*, *USP6NL*, *ECHDC3*, *PROSER2*, *UPF2*, *DHTKD1*, *SEC61A2*, *NUDT5*, *CDC123*, *CAMK1D*, *CCDC3*, *OPTN*, *MCM10*, *UCMA*, *PHYH*, *SEPHS1*, *BEND7*, *PRPF18*, *FRMD4A*, *FAM107B*, *CDNF*, *HSPA14*, *SUV39H2*, *DCLRE1C*, *MEIG1*, *OLAH*, *ACBD7*, *C10orf111*, *RPP38*, *NMT2*, *FAM171A1*, *ITGA8*, *MINDY3*, *PTER*, *C1QL3*, *RSU1*, *CUBN*, *TRDMT1*, *VIM*, *ST8SIA6*, *HACD1*, *STAM*, *TMEM236*, *MRC1*, *SLC39A12*, *CACBN2*, *NSUN6*, *ARL5B*
3	rea(1)	chr1:3,290,001–3,297,000	0.007	*PRDM16*
		chr1:7,682,001–7,685,000	0.003	***CAMTA1***
		chr1:12,030,001–12,898,000	0.87	*PLOD1*, *MFN2*, *MIIP*, *TNFRSF8*, *VPS13D*, *DHRS3*, *AADACL4*, *C1orf158*, *PRAMEF12*, *PRAMEF1*, *PRAMEF11*
		chr1:14,176,001–14,443,000	0.27	*-*
		chr1:27,415,001–27,452,000	0.04	*SLC9A1*
		chr1:236,176,001–236,183,000	0.007	*NID1*
		chr1:246,031,001–246,033,000	0.002	*SMYD3*

Bold genes indicate known disease-causing OMIM genes explaining all or part of the clinical phenotype.

**Table 2:** Overview of breakpoints and genomic regions involved in Case 1 and Case 2.

Case	Cytogenetic aberration	Type of SV	ChrA	PosA	ChrB	PosB	MH	Insertion	Repeat
1	t(2;8;15)(q34;q23.3;q21.3)	Translocation	15	55083061	2	209425831	-	-	L1M5/MLT1E1
		Inversion	2	211567929	2	211580840	-	G	L2/-
		Translocation	15	55083064	2	211580844	T	-	L1M5/-
		Translocation	2	212551796	8	129040005	-	-	L1MD1/L2b
		Translocation	2	214880375	8	114508085	-	-	MER72/ERVL-B4-int
2	t(1;10;5)(q31.3;p12.31;q23.2)	Translocation	1	196997343	5	124956736	-	-	L1MA9/-
		Translocation	1	196997344	10	20816166	-	-	AluJr/L1PA4
		Translocation	10	20816168	5	124956731	TA	-	L1PA4/-
		Deletion	10	4689760	10	19120882	TCA	-	-/-

Genomic positions are given in kb from the p telomere (Hg19). The microhomology (MH) column indicates the presence of microhomology between position A and B, while the insertion column describes any inserted sequence. The repeat column presents any repeat found within 100 bp of the breakpoints that could have mediated the formation of the derivative chromosome. The UCSC repeat masker was used to determine the position of repeats throughout the genome. Chr: chromosome, Pos: position, SV: structural variant, MH: microhomology

A total of eight breakpoints and five breakpoint junctions were identified in Case 1. Three out of five of the breakpoint junctions were detected using all four WGS technologies. The 12 kb inversion on chromosome 2 (fragment C, Fig 1A) was detected using both short-read sequencing (PCR-free PE and MP) technologies and the linked-reads technology with the Supernova pipeline, but not by optical mapping likely due to its small size. One of the translocation junctions between chromosome 2 and chromosome 8 was not detected using the linked-reads technology (Supernova pipeline). None of the breakpoint junctions in Case 1 were found with the linked-reads technology using the Long Ranger pipeline.

Finally, eight breakpoints were located within repeats elements. Sequence homology was found in two out of these junctions, however only short stretches of microhomology indicate that no fusion repeat elements were formed (Table 2, S2 Fig).

### Case 2

The t(1;5;10) complex rearrangement was first identified using regular karyotyping and FISH. A large deletion of 14.4 Mb located at chromosome 10p12 was not detected by the initial chromosome analysis, possibly due to the fact that multiple chromosomal segments were involved and the deletion was located in the translocation breakpoint region. Instead, the deletion was identified during characterization of the rearrangement using FISH and later confirmed by aCGH. The molecular cytogenetics data was reported previously [15]. WGS confirmed the cytogenetic findings (Fig 1B), including the deletion (segment F, Fig 1B) that involved 75 protein-coding genes (Table 1). A total of five breakpoints and four breakpoint junctions were identified. Breakpoint junctions revealed 0 to 3 nt of microhomology, and no SNVs or insertions neither in the junctions or flanking the junctions (Table 2, S2 Fig). Three out of five breakpoints of this complex translocation were located within repeat elements, but no evidence for fusion repeats elements were observed in the breakpoint junctions, meaning that the rearrangement produced a truncated repeat (S2 Fig). The breakpoints of the large deletion on chromosome 10 were both located far from any repeat element (Table 2). Using the WGS data, the molecular karyotype could be determined to be t(1;10;5)(q31.3;p12.31;q23.2) seq[GRCh37] g.[chr1:pter_cen_196997343::chr5:124956736_qter] g.[chr5:pter_cen_124956731::chr10: 20816168_pter] g.[chr10:qter_cen_20816166::4689760_19120882del::chr1:196997343_qter]. No other rare structural variants were identified in the microarray or WGS data. All breakpoint junctions indicated by the cytogenetic analysis were found by all four WGS technologies applied, although only PCR-free PE WGS and linked-read sequencing resolved the junctions on the nucleotide level.

### Case 3

**Fig 2:**
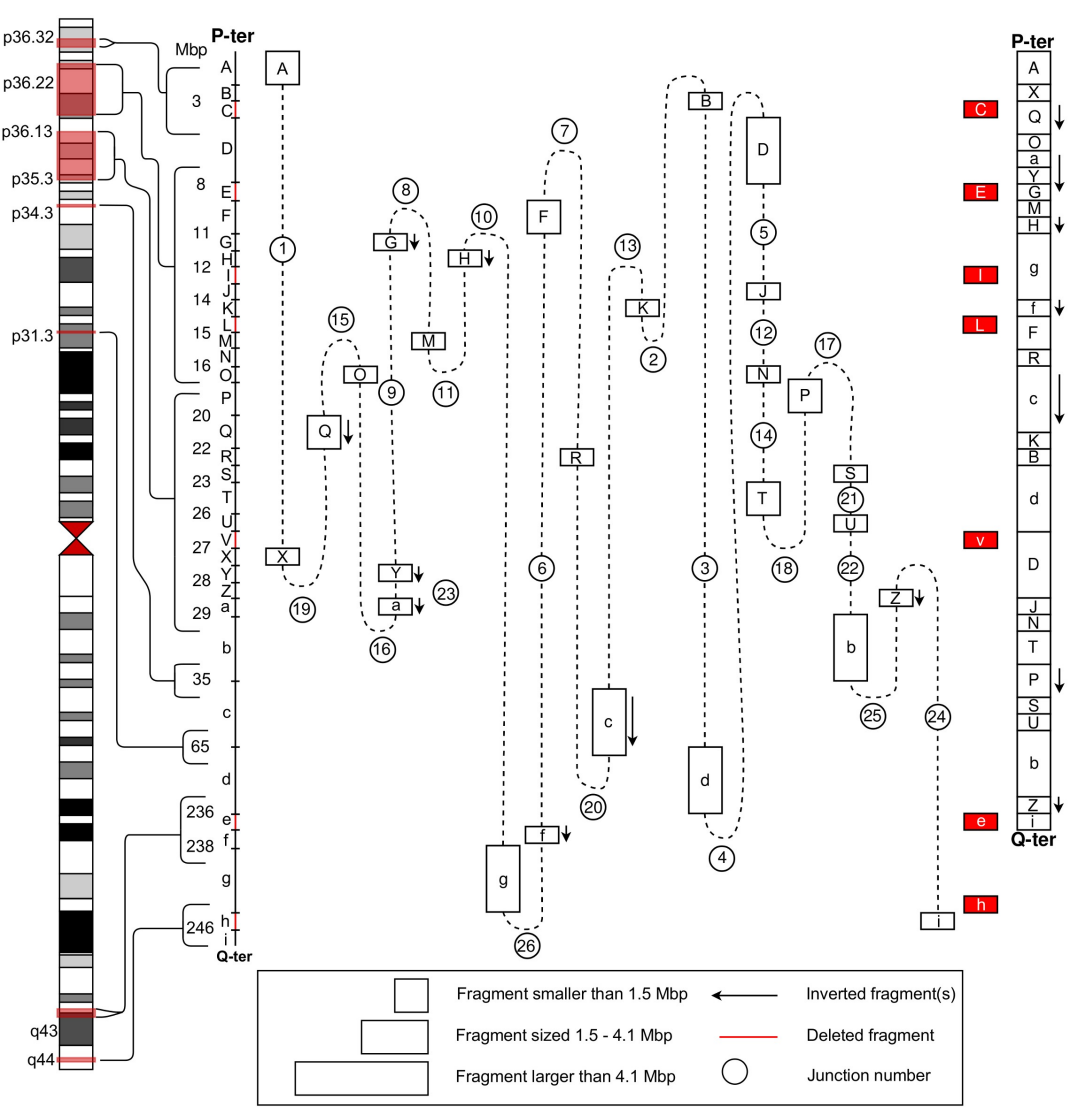
Molecular characterization of an extremely complex intrachromosomal rearrangement of chromosome 1. Whole genome sequencing enabled mapping of the rearranged chromosome 1. Chromosome 1 had been disrupted in 33 positions, and puzzled back together with deletions or inversions of some fragments. Junction numbers and characteristics for each junction are listed in Table 3. On the left side is a schematic of human chromosome 5 with red boxes indicating regions containing breakpoints. The 34 chromosomal fragments labeled from A-i are shown as a vertical linear diagram with fragment size given in Mb. In the middle the whole genome sequencing results are illustrated. Links between fragments are shown as dashed lines and the junction numbers are given. The final rearranged chromosome is shown on the right with deleted fragments in red and inversions indicated by an arrow.

First, karyotyping identified an unusual banding pattern on the short arm of chromosome 1. Follow-up analysis using FISH and BAC array identified 14 breakpoint junctions including a 0.87 Mb deletion located at 1p36.2, which was previously reported in Lindstrand et al. (2008) [16]. Combined analysis of the four different WGS technologies confirmed those 14 breakpoint junctions and unveiled 12 additional ones (Table 3) originating from chromosomal segments translocated, inverted, and deleted involving both the short and the long arm of chromosome 1 (Fig 2). Hence, a total of 33 breakpoints and 26 breakpoint junctions were identified after WGS analysis. The WGS analysis identified seven deletions at the rearrangement breakpoints (Table 1). In the breakpoint junctions, microhomology was found in 10 junctions (2–6 nt), and one junction contains a 9 bases long non-templated insertion (Table 3, S2 Fig). Every breakpoint junction contained at least one repeat region, mostly *Alu* elements (Table 3). The junctions that do not involve *Alu* elements are located within a wide range of distinct types of repeat regions. The second major group of repeats were found to be LINE elements (11 breakpoints), remaining breakpoints seem to be scattered randomly amongst various repeats including simple repeats, LTR elements, and retroviral elements (Table 3).

**Table 3:** Overview of breakpoints and genomic regions involved in Case 3.

Jct	Start	End	MH	Insertion	Repeat	Gene brpA	Gene brpB
1	2581232*	27452000	N.i.	N.i.	AluSg/AluSz6	*TTC34*	***SLC9A1***
2	2684269*	14176001	N.i.	N.i.	-/THE1B	*TTC34*	-
3	3290905	64810516	G	-	-/L3	***PRDM16***	-
4	3296814	236176103	CT	-	(CACCC)n/-	***PRDM16***	*NID1*
5	7682335	12897986	-	-	MIRb/AluYa5	***CAMTA1***	*-*
6	7685352	236183504	CCTCTT	-	-/AluSx	***CAMTA1***	*NID1*
7	10775956	21736058	-	GGTTTAAAC	(CACCC)n/AluY	*CASZ1*	-
8	10775956	14443177	-	-	(CACCC)n/THE1B-int	*CASZ1*	-
9	11950223	27642518	C	-	LTR5B/AluSz6	-	-
10	11950229	237674299	C	-	LTR5B/-	-	***RYR2***
11	12029894	14870594	GT	-	AluJb/-	***PLOD1***	-
12	13975090	14870595	-	-	L1MC5/-	-	-
13	13975102	35045018	-	-	L1MC5/MER41B	-	-
14	16136097	23235619	-	-	AluSz6/-	-	*EPHB2*
15	16136098	20149345	-	-	AluSz6/L4	-	*-*
16	16147000	29030516	-	-	AluSx/AT_rich	-	*GMEB1*
17	16147001	23117213	C	-	AluSx/-	-	*EPHB2*
18	20149331	26177921	-	-	L4/L1MB7	-	*AUNIP*
19	21736058	27642518	C	-	AluY/AluSz6	-	-
20	23117213	64810516	-	-	-/L3	-	-
21	23235618	26177922	-	-	-/L1MB7	*EPHB2*	*AUNIP*
22	27414473	29030516	GGGC	-	ERVL-E-in/AT_rich	-	*GMEB1*
23	27667160	28083088	-	-	AluY/AluSx1	-	*FAM76A*
24	27667279	246033001	-	-	AluY/-	-	*SMYD3*
25	28083088	35045017	-	-	AluSx1/MER41B	*FAM76A*	-
26	237674294	246030822	-	-	-/L2a	***RYR2***	*SMYD3*

Genomic coordinates are given in Hg19. The microhomology (MH) column indicates the presence of any microhomology between the start and end positions, while the insertion column describes any inserted sequence. The repeat column presents any repeat found within 100 bp of the breakpoints. OMIM disease related genes are shown in bold text. Jct: junction, MH: microhomology, N.i.: no information * = estimated breakpoint

**Fig 3:**
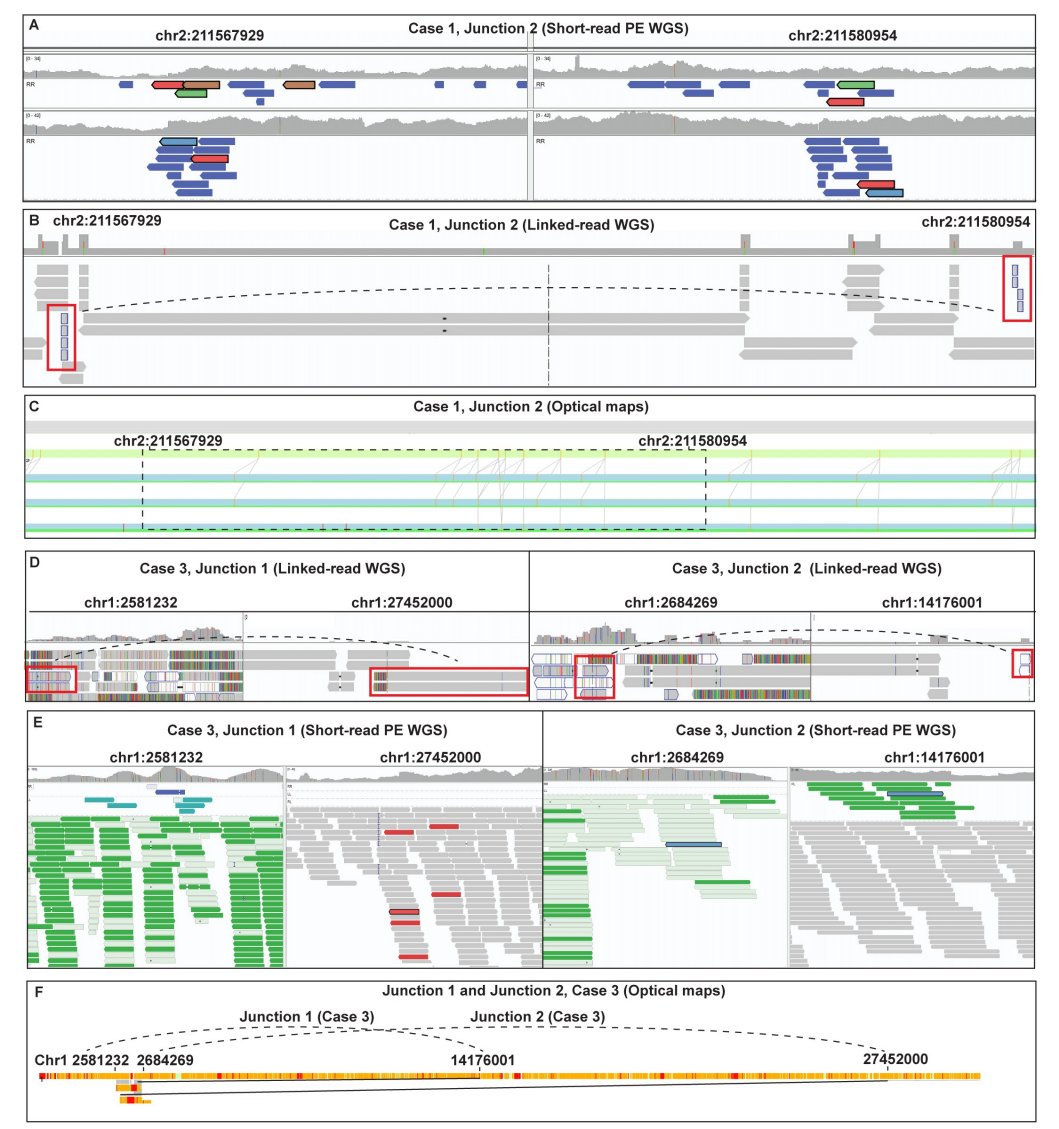
Detection rate mainly differs between the technologies with small SVs and within repetitive regions. In the analysis of Case 1 a small 13kb inversion on chromosome 2 (fragment C, Fig 1; Case 1, junction 2, Table 4) was detected using short-read WGS (PE and MP) **(A)** and linked-read WGS **(B),** but not using optical maps **(C)**. In A and B, paired or linked reads in opposite directions are present on each side of the junction and in C, the links between the optical maps are aligned correctly to the reference. In contrast, two breakpoint junctions from Case 3 (Junction 1 and 2, Table 3) were not detected using short-read WGS (PE or MP) **(D)**, but was only seen with linked-read WGS **(E)** and optical mapping **(F)**, due to repetitive regions in one of the breakpoints. In D and E, linked and paired reads are present on one side of the junctions, and on the other side are several poorly annotated reads with an unexpectedly high coverage, indicative of repetitive regions. In F, the optical maps are split at positions chr1:2581232 and chr1:2684269 (left), and the same maps continue at chr1:14176001 and chr1:27452000, respectively (right). The short-read WGS data in A, B, D and E is visualized in IGV and the optical maps data in C and F is from Bionano access.

In total, 26 breakpoint junctions were detected in Case 3 using combined analysis. Two junctions, junction 1 and junction 2, were not detected using any short-read (PCR-free PE and MP) method, likely because they map within low-copy repeats (LCRs) in a 125 kb large intron of *TTC34*. Breakpoints located within such regions may require longer reads to be correctly mapped, and indeed only optical mapping and linked-read sequencing were able to detect those junctions (Fig 3, Table 4). Of note, all 26 breakpoint junctions were detected using the linked-read WGS technology, although we were required to use both the Long Ranger and Supernova pipelines as they complement each other (Table 4). PCR-free PE WGS detected 24 out of 26 breakpoint junctions, all of them supported by split reads. MP WGS identified 23 out of 26 breakpoint junctions but none of them was solved on the nucleotide level as expected for MP, whereas optical mapping detected 20 out of 26 breakpoint junctions (Table 4).

**Table 4:** Detailed results from the comparison of different sequencing technologies.

		Breakpoint junctions				Short-read PE		Short-read MP		Optical maps		Linked-read Long Ranger		Linked-read Supernova	
Case	Jct	ChrA	Start	ChrB	Stop	Start chrA	Stop chrB	Start chrA	Stop chrB	Start chrA	Stop chrB	Start chrA	Stop chrB	Start chrA	Stop chrB
1	1	15	55083064	2	209425831	55083064	209425831	55083064	209425759	55089032	209402341	N.i.	N.i.	55083064	209425831
	2	2	211567929	2	211580954	211567929	211580954	211567929	211580954	N.i.	N.i.	N.i.	N.i.	211567929	211567929
	3	15	55083060	2	211580725	55083060	211580725	55074707	211580771	55065367	211588781	N.i.	N.i.	55083060	211580725
	4	2	212551644	8	129040042	212551644	129040042	212551653	129040144	212542133	129041041	N.i.	N.i.	212551644	129040001
	5	2	214880375	8	114508085	214880375	114508085	214886416	114500483	214907985	114499568	N.i.	N.i.	N.i.	N.i.
2	1	1	196997343	5	124956736	196997343	124956736	196994135	124956869	196978354	124976031	196997346	124956731	N.i.	N.i.
	2	1	196997432	10	20816222	196997432	20816222	197000507	20816587	197040164	20824882	196997340	20816229	N.i.	N.i.
	3	10	20816224	5	124956733	20816224	124956733	20812880	124958603	20804445	124913158	20816227	124956736	20816225	124956734
	4	10	4689832	10	19120882	4689832	19120882	4689419	19120882	4669757	19134619	19120884	4689840	N.i.	N.i.
3	1	1	2581232	1	27452000	N.i.	N.i.	N.i.	N.i.	2587901	27464776	N.i.	N.i.	2624548	27452144
	2	1	2684269	1	14176001	N.i.	N.i.	N.i.	N.i.	2684269	14173037	N.i.	N.i.	2625721	14175578
	3	1	3290905	1	64810516	3290905	64810516	3290638	64810985	3288732	64862703	N.i.	N.i.	3290905	64810516
	4	1	3296814	1	236176103	3296814	236176103	N.i.	N.i.	N.i.	N.i.	3295874	236176381	3296360	236176372
	5	1	7682335	1	12897986	7682335	12897986	7682260	12898340	7681134	12908586	7682180	12898137	N.i.	N.i.
	6	1	7685352	1	236183504	7685352	236183504	7689171	236183789	7685681	236193515	7685344	236183496	N.i.	N.i.
	7	1	10775956	1	21736058	10775956	21736058	10773838	21736071	10768488	21736601	10775947	14443168	N.i.	N.i.
	8	1	10775956	1	14443177	10775956	14443177	10780413	14443430	N.i.	N.i.	10775964	21736050	N.i.	N.i.
	9	1	11950223	1	27642518	11950223	27642518	11949895	27642567	11947447	28085587	11950233	27642509	N.i.	N.i.
	10	1	11950229	1	237674299	11950229	237674299	11952419	237674508	14864206	237679257	11950220	237674290	11950229	237674299
	11	1	12029894	1	14870594	12029894	14870594	12029206	14869373	N.i.	N.i.	12029904	14870604	12029894	14870594
	12	1	13975090	1	14870595	13975090	14870595	13974941	14871025	13973279	14872976	13975098	14870587	13975090	14870595
	13	1	13975102	1	35045018	13975102	35045018	13977983	35046971	13982091	35047391	13975094	35045018	13975102	35045018
	14	1	16136097	1	23235619	16136097	23235619	16135371	23235980	16129345	23237153	16136105	23235661	16136097	23235619
	15	1	16136098	1	20149345	16136098	20149345	16139274	20149848	N.i.	N.i.	16136089	20149366	16136098	20149345
	16	1	16147000	1	29030516	16147000	29030516	16146491	29025749	N.i.	N.i.	16147010	29030525	N.i.	N.i.
	17	1	16147001	1	23117213	16147001	23117213	16149609	23119367	16153803	23120068	16136105	23235661	16147001	23117213
	18	1	20149331	1	26177921	20149331	26177921	20149195	26174604	20123909	26163960	20149340	26177929	N.i.	N.i.
	19	1	21736058	1	27642518	21736058	27642518	21735951	27639797	21721930	27627697	21736067	27642527	N.i.	N.i.
	20	1	23117213	1	64810516	23117213	64810516	23117169	64806842	23116865	64808492	23117222	64810424	23117213	64810516
	21	1	23235618	1	26177922	23235618	26177922	23235543	26178144	23234405	26178892	23235614	26177914	23235618	26177922
	22	1	27414473	1	29030516	27414473	29030516	27413006	29030699	27400938	29032646	27414484	29030566	N.i.	N.i.
	23	1	27667160	1	28083088	27667160	28083088	27667122	28083292	N.i.	N.i.	27667283	28083081	27667278	28083088
	24	1	27667279	1	246033001	27667279	246033001	27669970	246033207	27673872	246031926	27667271	246033016	27667279	246033001
	25	1	28083088	1	35045017	28083088	35045017	28082851	35042558	28075475	35043873	28083097	35045020	N.i.	N.i.
	26	1	237674294	1	246030822	237674294	246030822	237674208	246027906	237673047	246031926	237674304	246030756	N.i.	N.i.

Genomic coordinates are given in Hg19. Chr: chromosome, Jct: junction, MP: mate-pair, PE: paired-end, N.i.: no information, indicates when the specific breakpoint junction could not be identified by the sequencing technology. Column “Breakpoint junctions” refers to the genomic coordinates for each junction as defined by split reads and further confirmed by PCR/Sanger sequencing.

### General method comparison for solving complex chromosomal rearrangements

Comparing the results of the different technologies, it was found that all technologies tested here could detect the majority of the junctions (Tables 4 and 5). PCR-free PE WGS and the linked-read technology present the highest detection rate: PE detected all but two junctions whereas linked-reads detected all but one junction. Moreover, both PCR-free PE WGS and linked-read WGS share the highest resolution (1 bp). MP WGS present a resolution of ~400bp with a false-negative rate of 9% (fails to report three junctions out of the total 35 junctions) (Table 5). Lastly, optical mapping has the lowest detection rate since it fails to report seven junctions of the total 35 junctions (20%) and the lowest resolution of ~ 7 kb (Table 5).

**Table 5:** General comparison of the sequencing technologies.

Technology	Number of junctions detected/Total (Split reads)		
	Case 1	Case 2	Case 3	Resolution
Short-read (PE)	5/5 (5)	4/4 (4)	24/26 (23)	1bp
Short-read (MP)	5/5 (0)	4/4 (0)	23/26 (0)	400bp
Optical maps	4/5 (4)	4/4 (4)	20/26 (20)	7.2kb
Linked-read	4/5 (4)	4/4 (4)	26/26 (26)	1bp

PE: paired-end, MP: mate-pair. The total number of junctions detected indicates the amount of breakpoint junctions related to the complex chromosomal rearrangement that were detected from the total amount of structural variants for each method. The total number of breakpoint junctions supported by split reads is indicated within parentheses. Resolution is the median distance between the generated calls and the exact breakpoint position.

### Delineating and identifying derivative chromosomes from WGS data

The three CCRs presented here were previously identified using chromosome analysis, therefore the WGS analysis was focused on delineating the structure of the derivative chromosomes. However, to simulate a “WGS first” scenario and evaluate the utility of each of the techniques applied here for SV detection without a prior hypothesis, we ran FindSV developed for PCR-free PE and MP WGS data. The FindSV analysis pipeline is described in the WGS methods section. Generated calls were thereafter ranked based on i) frequency in the SweGen SV database [17] ii) amount of discordant read pairs and iii) size in base pairs, or chromosomal position. Based on these filtering criteria, calls pinpointing the breakpoints of the complex rearrangements were ranked high in the generated lists of rare SVs in all cases (S1 Table). The FindSV pipeline generated 162 calls from the PCR-free PE data in Case 3 from which 34 well-supported interchromosomal calls were ranked above the intrachromosomal rearrangement on chromosome 1 (S1 Table). These calls are most likely due to mobile elements [18], therefore we concluded that filtering of PCR-free PE data needs to be optimized to minimize false-positive interchromosomal calls due to repetitive elements and favor ranking of potentially false-negative intrachromosomal rearrangements. Lastly, not all breakpoint junctions will be detected in the FindSV output data. FindSV may fail to detect breakpoint junctions that are located in repetitive regions as well as regions that are poorly covered due to various technical artifacts or low DNA quality. All highly ranked calls are manually inspected in IGV, which allows for detection of additional breakpoints and small aberrations in the junctions.

Analysis of the linked-read and optical map pipelines in a simulated “WGS-first” scenario was more challenging because both technologies produce a large number of calls (>1000). In addition, there is still a lack of frequency databases for these technologies, which makes filtering and ranking based on public database frequencies currently not possible. We concluded that using optical maps or linked-read sequencing technologies as the initial screening techniques for CCRs might not be feasible with current pipelines until more frequency data is available. Furthermore, optical mapping has a lower resolution limitation which hamper its use to detect smaller and more complex junctions, including the small 12 kb inversion of fragment C in Case 1 (Fig 3A–3C).

Importantly, however, we found that optical mapping and linked-read WGS performs better than short-read WGS in highly repetitive regions or paralogous regions (such as Junctions 1 and 2, Case 3, located within LCRs). Example of the junctions that could not be detected using any of the short-read WGS protocols is shown in Fig 3D and Fig 3E.

### Sensitivity of WGS SV calling compared to CNVs detected by aCGH

To compare the utility of all WGS technologies used here, we also performed a comparison of all polymorphic CNVs present in the three probands of the present study detected with aCGH (S2 Table). In brief, CNV calls obtained by WGS were compared to those from two different aCGH platforms: a custom designed 400K genome-wide array with ~ 2000 targeted high-resolution genes and a commercial 1M medical exon array from OGT with ~5000 targeted high-resolution genes. The array calls that were also found with at least one of the WGS methods were considered confirmed and hence more likely to be true variants. The sensitivity for detecting such confirmed CNVs in each case and method is given in S2 Table and the summarized sensitivity per method in Table 6. The data suggest that PCR-free PE WGS has a high detection rate on both small (<0.1 kb) and large (>10 kb) SVs and offers the highest overall detection rate. Linked-read WGS technology has the second highest detection rate, mainly because of the Supernova pipeline and the Long Ranger small deletions algorithm. Lastly, MP WGS and optical mapping perform similarly: both technologies perform well on the larger CNVs, but fail to detect a large number of smaller variants (S2 Table). Overall, it is clear that the high resolution of linked-reads WGS and PCR-free PE WGS allows for the detection of small SVs that MP WGS and optical mapping does not have the required resolution to detect.

**Table 6:** Comparison of the four WGS technologies versus array comparative genomic hybridization.

**Method**	**Case 1 custom 400K array**				**Case 2 custom 400K array**				**Case 3 custom 400K array**			
	Total	Del	Dup	Sens	Total	Del	Dup	Sens	Total	Del	Dup	Sens
aCGH	83	18	65	-	32	17	15	-	60	11	49	-
aCGH (confirmed variants)	17	10	7	1	14	8	6	1	14	11	3	1
Bionano	6	2	4	0.35	6	3	3	0.43	4	1	3	0.29
Bionano BssSI	5	1	4	0.29	4	3	1	0.29	3	1	2	0.21
Bionano BspQI	4	2	2	0.24	5	2	3	0.36	2	1	1	0.14
Illumina PE	13	9	4	0.76	11	6	5	0.79	13	11	2	0.93
Illumina MP	10	7	3	0.59	4	1	3	0.29	5	4	1	0.36
10X linked reads	6	4	2	0.35	8	6	2	0.57	9	8	1	0.64
10X linked reads*	1	0	1	0.06	2	1	1	0.14	3	2	1	0.21
10X linked reads**	4	4	0	0.24	6	5	1	0.43	6	6	0	0.43
10X linked reads***	3	2	1	0.18	5	5	0	0.36	5	5	0	0.36
	**Case 1 1M array**				**Case 2 1M array**				**Case 3 1M array**			
aCGH	126	60	66	-	186	143	43	-	129	26	103	-
aCGH (confirmed variants)	27	20	7	1	19	18	1	1	24	17	7	1
Bionano	12	7	5	0.44	6	6	0	0.32	11	6	5	0.46
Bionano BssSI	6	3	3	0.22	4	4	0	0.21	9	4	5	0.38
Bionano BspQI	9	6	3	0.33	4	4	0	0.21	6	3	3	0.25
Illumina PE	21	17	4	0.78	15	15	0	0.79	17	14	3	0.71
Illumina MP	13	11	2	0.48	10	9	1	0.53	10	7	4	0.42
10X linked reads	9	8	1	0.33	13	13	0	0.68	11	10	1	0.46
10X linked reads*	1	0	1	0.04	1	1	0	0.05	2	1	1	0.08
10X linked reads**	5	5	0	0.19	8	8	0	0.42	8	8	0	0.33
10X linked reads***	7	7	0	0.26	11	11	0	0.58	9	9	0	0.38

Total number of detected CNVs, deletions and duplications for each case and technology/pipeline.

*Long Ranger—large SV

** Long Ranger—deletions

*** Supernova. Del: deletion, Dup: duplication, Sens: sensitivity. The aCGH row (confirmed variants) indicates the number of aCGH variants that were confirmed with any of the four WGS technologies. Each sample was analyzed using two arrays: a custom 400K array, as well as a commercial medical exome 1M array.

### Comparison of genome wide SV calls from different WGS technologies

Next, we used the Case 2 WGS data, which were of the best quality among the three cases, to compare the sensitivity, number of calls, reported variant sizes, and reported variant types of the WGS technologies to one-another. Using SVDB (https://github.com/J35P312/SVDB) [10], we found that the technologies differ regarding the overlap of the variant calls (Table 7). In particular, optical mapping and the Illumina based technologies detect only a few hundred (6.6%) overlapping calls (Table 7). In contrast, MP and PCR-free PE WGS produce the largest fraction of overlapping calls with nearly 71% of the MP calls also detected by PCR-free PE WGS. The overlap between PCR-free PE WGS and linked-reads WGS was 42% (Table 7).

**Table 7:** Matrix table comparing the amount of genome wide overlapping SV calls detected by four WGS technologies in Case 2.

	Optical maps	PE WGS	MP WGS	Linked-reads
**Optical maps**	10622 (100%)	-	-	-
**PE WGS**	318 (6.6%)	4802 (100%)	-	-
**MP WGS**	229 (36.6%)	626 (70.9%)	883 (100%)	-
**Linked-reads**	164 (1.5%)	2007 (41.8%)	185 (21%)	16718 (100%)

WGS: whole genome sequencing, MP: mate-pair, PE: paired-end, within parentheses: each pairwise comparison given as percentages of overlapping SV calls.

**Fig 4:**
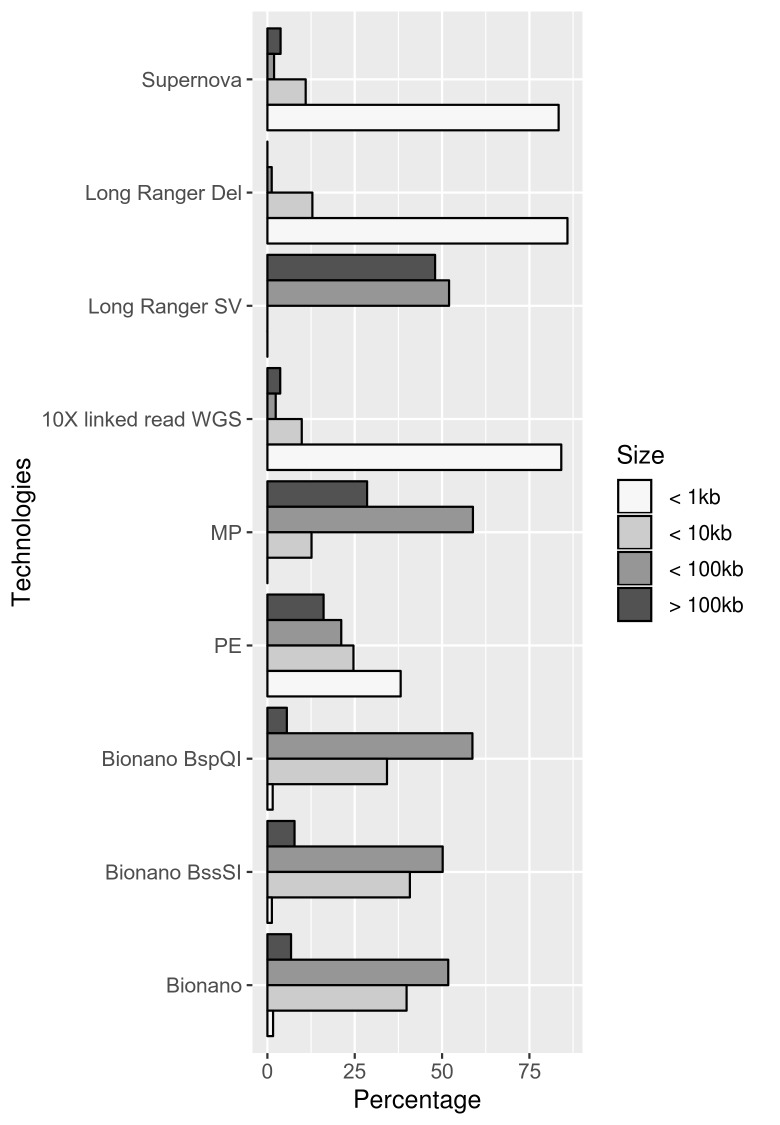
Percentages of the amount and type of structural variant (SV) calls generated by each technology and analysis pipeline, based on size. Approximately 90% of calls from Bionano optical mapping are between 10–100 kb in size and few calls are larger than 100 kb or smaller than 1 kb. Calls from paired-end (PE) WGS data is quite evenly spread, with slightly more calls <1 kb. The structural variant calls from the mate-pair (MP) WGS are sized between 10–100 kb. The linked-read WGS data was analyzed using three algorithms with different strengths, the Supernova *de novo* assembler, the Long Ranger large SV algorithm and the Long ranger deletion (Del) algorithm. Combining all callers, the linked-read WGS data produce a very high number of calls <1 kb.

There are many reasons why the number of overlapping calls may differ between technologies, including artifacts and features specific to each technology and calling pipeline that can influence the output (S2 Table). Bias towards variant types is also observed, for instance optical maps report no duplications and 6733 insertions while PE WGS reports 454 duplications but no insertions (Table 8). Moreover, due to distinct resolutions, each methodology also present a bias towards the SV sizes it detects. For instance, linked-read WGS and PCR-free PE WGS produce a large fraction of small variant calls (<0.1 kb), in contrast, most of the optical mapping and MP calls are sized 10–100 kb (Fig 4).

**Table 8:** Number of calls per variant type.

	Total	Del	Dup	Ins	Inv	Break-end	Unknown
**Bionano**	11147	3050	0	7109	84	904	0
**Bionano BssSI**	7013	1914	0	4342	64	693	0
**Bionano BspQI**	5480	1501	0	3482	29	458	0
**PE WGS**	4802	2310	454	0	373	1665	0
**MP WGS**	883	494	279	0	29	81	0
**10X linked-reads**	16718	8596	726	484	1140	5762	10
**Long Ranger SV**	211	26	5	0	4	166	10
**Long Ranger Del**	3963	3933	0	0	0	36	0
**Supernova**	14495	6480	725	484	1138	5668	0

Break-end indicates intra- and interchromosomal translocations.

PE: paired-end, MP: mate-pair, SV: structural variant, Del: deletion, Dup: duplication, Ins: insertion, Inv: inversion

Next, we compared the overlap between the variant calls and three public datasets: the Database of Genomic Variants (DGV) [19], the HG002 integrated call-set compiled by the Genome In A Bottle consortium (GIAB) (https://github.com/genome-in-a-bottle) [20], as well as the CNV list published in a previous paper by Conrad et al. (2010) [21] (Table 9). Notably, the performance between the technologies differs depending on which database they are compared to. Comparing the methodologies used here to the CNVs reported in Conrad et al. [21], we found that Bionano and PCR-free PE WGS achieve similar numbers. MP WGS reports fewer variants matching the CNVs listed in Conrad et al, and linked-read WGS detect only a few of those CNVs (Table 9).

**Table 9:** Comparison between the WGS technologies and three public datasets.

**Conrad et al. (2010)**						
**Technology**	**Del**		**Dup**			
Bionano	43		88			
Bionano BssSI	34		61			
Bionano BspQI	21		48			
PE WGS	43		94			
MP WGS	26		69			
10X linked reads	14		15			
Long Ranger SV	2		3			
Long Ranger Del	3		2			
Supernova	11		14			
Total	6960		4740			
**DGV**						
**Technology**	**Complex**	**Del**	**Dup**		**Inv**	**Ins**
Bionano	83	1780	1000		84	586
Bionano BssSI	60	1236	651		58	377
Bionano BspQI	53	1032	613		53	390
PE WGS	78	2670	1036		167	96
MP WGS	45	459	437		40	51
10X linked reads	50	4438	707		78	135
Long Ranger SV	19	46	40		13	9
Long Ranger Del	16	2787	314		10	16
Supernova	35	3374	575		61	115
Total	578	258436	81122		2652	36577
**HG002 integrated call-set**						
**Technology**	**Del**		**Ins**			
Bionano	58		0			
Bionano BssSI	43		0			
Bionano BspQI	35		0			
PE WGS	1128		4			
MP WGS	79		0			
10X linked reads	1859		8			
Long Ranger SV	3		0			
Long Ranger Del	1467		1			
Supernova	1445		1			
Total	28773		30576			

Del: deletion, dup: duplication, inv: inversion, ins: insertion, SV: structural variant, MP: mate-pair, PE: paired-end. The Total row indicates the total number of variants of each type in the three datasets: DGV [19], the HG002 integrated call-set [22] and Conrad et al. (2010) [21]. BssSI and BspQI are the two restriction enzymes used in the Bionano optical mapping experiment, Long Ranger: the 10X Genomics mapping-assembly based pipeline, Supernova: the results of a custom pipeline utilizing the Supernova *de novo* assembler.

In contrast, linked-read WGS performs better on the DGV database, and detects the largest number of deletions (Table 9). Despite the high detection rate of these deletions, linked-read WGS performs relatively poor on all other variant types. Similar to the comparison of the Conrad et al. dataset [21], optical maps and PCR-free PE WGS report nearly the same amount of variants. However, optical maps detect a greater number of insertions, while PCR-free PE WGS detects a greater number of deletions.

MP WGS detects the smallest number of variants, however, most of the MP variant calls do match a DGV variant indicating that the precision of MP WGS is high. In contrast, a smaller fraction of optical maps and Long Ranger calls are similar to DGV variants, indicating a lower precision of these technologies.

Lastly, the technologies were compared to the HG002 (GIAB) integrated call-set, which consists of mainly small SVs (<0.1 kb) (S4 Table) detected using both long- and short-read sequencing methods (ftp://ftp-trace.ncbi.nlm.nih.gov/giab/ftp/data/AshkenazimTrio/analysis/NIST_SVs_Integration_v0.6/README_SV_v0.6.txt). It was found that linked-read WGS and PCR-free PE WGS detect the largest number of those variants, while optical maps and MP WGS produce relatively few hits. These results are not surprising given the resolution and size bias that those methodologies present (Table 5).

## Discussion

It has previously been demonstrated that cytogenetically balanced CCRs often contain cryptic deletions in the breakpoints, explaining the clinical phenotype of the carrier patients in many cases [5]. Here we report two individuals with complex interchromosomal translocations (Case 1 and Case 2) and one individual with a very complex intrachromosomal rearrangement (Case 3) for whom we used multiple combined cytogenetic and molecular methodologies to refine the alterations in their genome content which aided clinical assessment.

In Case 1, a 5 Mb deletion on chromosome 2 and a 14.5 Mb deletion on chromosome 8 was identified using FISH mapping. Further analysis using aCGH and WGS, showed that the 5 Mb deletion actually consisted of two deletions, 2.1 Mb and 2.3 Mb, respectively. WGS could also demonstrate that a 970 kb genomic segment, originally located in-between the two deletions, had been translocated onto chromosome 15. The deletion on chromosome 8 in Case 1 covers the TRPSII (Trichorhinophalangeal syndrome type II, TRPSII, Langer Gideon Syndrome, LGS, MIM# 150230) locus and most of the characteristic symptoms of TRPSII were present in Case 1.

In Case 2, the translocated piece of chromosome 10 contained a 14.5 Mb deletion, encompassing the typical region for hypoparathyroidism, sensorineural deafness, and renal disease syndrome (HDRS, Barakat syndrome, MIM# 146255), characterized by the triad hypoparathyroidism, renal dysplasia and hearing loss. The common cause of HDR syndrome is mutations in *GATA3* [23]. Deletions of 10p are recurrent, and *GATA3* has been pinpointed as the causative gene for HDR syndrome seen in 10p deletions. The size and location of 10p deletions vary, as well as the clinical picture [15].

Case 3 presented with mild dysmorphic features, psychomotor delay, ectopic left kidney, minor hearing disability, dysphasia, feeding difficulties, mild short stature, and developmental delay. Thorough analysis of the 26 breakpoints on chromosome 1 revealed six known disease-related OMIM disease genes to be disrupted or affected by a deletion (*RYR2*, *MFN2*, *CAMTA1*, *SLC9A1*, *PRDM16* and *PLOD1*). Two of the OMIM genes (*CAMTA1* and *PRDM16*) were identified using FISH in a previous study of the same case, while remaining genes were novel findings using WGS [16]. *CAMTA1* is known to cause autosomal dominant cerebellar ataxia (non-progressive) with mild intellectual disability (MIM# 614756), with phenotypes including delayed psychomotor development, cerebellar ataxia, intellectual disability, neonatal hypotonia, and variable dysmorphic features, some of them consistent with the phenotype in Case 3. Remaining genes have not been associated with any phenotypes present in Case 3 and the full phenotype (ectopic kidney, hearing disability) in this individual could not fully be explained by the WGS analysis, but needs further investigation of the genes affected by the rearrangement.

There are a number of events that could lead to complex rearrangements, including replication-based mechanisms (fork-stalling and template-switching (FoSTeS) model) [24] and chromothripsis [25]. WGS allowed for detailed analysis of all breakpoint junctions on the nucleotide level in Case 1 and Case 2, and all junctions but one in the two complex translocations were blunt, with maximum of two nucleotides microhomology. In one breakpoint junction, there were two small insertions of three and four nucleotides, respectively. They did not seem to be duplicated or templated from nearby sequences, which would have indicated a replicative error mechanism, instead the mutational signatures in both Case 1 and Case 2 indicate non-homologous end-joining (NHEJ), characteristic of chromothripsis rearrangement junctions. The characteristics of the breakpoint junctions in Case 3 makes it likely that the complex rearrangement of chromosome 1 formed through a single catastrophic event. These characteristics include randomness of the DNA fragment joins, the DNA fragments appear to be randomly joined in inverted/non-inverted orientation, and the ability to walk along the derivative chromosome [26]. However, the rearrangement does not include a regularity of oscillating copy-number states [26], but only involves a small number of randomly spread deletions. In addition, the breakpoints of the q and p arms are separated by a 171 Mb DNA fragment, hence the breakpoints are not clustered as would be expected [26]. Further, the p and q arms of chromosome 1 seem to have been brought close together in a ring-like formation. Possibly, the p and q arm were brought together before the scattering of the chromosome, otherwise, the fragments of one arm would either be lost, or the fragments would be less prone to translocate between the arms. Hence, the rearrangement of chromosome 1 in Case 3 could have arisen either from the halted formation of a ring chromosome, or even through a chromothripsis event of a ring chromosome.

The four WGS technologies performed were utilized in three different settings: i) for solving the derivative chromosome structure of the three CCRs, ii) for a comparison of detection rate of polymorphic CNVs first detected by aCGH in the three cases and iii) for a general assessment of genome wide SV calls from the three cases as compared to calls present in the public datasets [19, 21, 22].

First, we found that no technology provides a significantly higher detection rate than the others regarding the ability to detect and solve the structure of the *de novo* CCRs presented herein. This is partly a result of the relatively small number of CCR junctions in this study (35), but also due to the high detection rate of all four WGS technologies. Moreover, although those CCRs are complex in nature, the majority of the breakpoint junctions could be uniquely mapped to regions that do not present complex repetitive genomic patterns such as LCRs, satellites, centromeric or telomeric repeats. This is exemplified for LCR-containing junctions 1 and 2 of Case 3 that could only be resolved by optical mapping and linked-read WGS. Those technologies require longer DNA molecules and therefore are more appropriated to resolve repeats than short reads. These regions are also more prone to rearrangements [27], and hence the ability to resolve junctions mapping within those structures is of great importance.

Although the detection rates of these methods are similar, the resolutions differ. Both PCR-free PE WGS and linked-read sequencing can resolve most breakpoints to base-pair resolution. In contrast, optical mapping provides the lowest resolution (~7 kb), which likely explains why it failed to detect breakpoints involving the smaller fragments of Case 1 and Case 3.

To assess whether the CCRs would have been detected in a “WGS-first” scenario the MP and PE WGS data was filtered based on allele frequency, but filtered variant lists of the linked-read and optical mapping calls could not be obtained as there are no frequency databases available for those technologies. Given the low similarity between PCR-free PE WGS and these two methods, the databases such as the SweGen cohort [17] or 1000 Genomes [28] would be of very limited use. Hence, in order to make linked-read WGS and optical mapping usable in a clinical setting, large populations would need to be sequenced using these methods, and the data made available through frequency databases.

Finally, comparing the four technologies to polymorphic CNVs, it was found that PCR-free PE WGS provides the highest sensitivity, closely followed by linked-read sequencing. MP WGS and optical mapping performed similarly, with almost half the detection rate of PCR-free PE WGS. Notably, both these two methods failed in detecting smaller CNVs (<10 kb). Furthermore, PCR-free PE WGS did find a significant number of CNVs that linked-read WGS failed to detect: these CNVs did either belong to Case 1, having partially degraded DNA and too short molecules to be sequenced by linked-read WGS, or the CNVs were subsequently detected using CNVnator instead, which is a read-depth caller and able to detect variants as small as 2 kb [29]. These variants exemplify that the large amounts of high performing Illumina WGS callers provide an edge over these more recent methods, whose pipelines are less mature, and still undergoing rapid development.

Lastly, the technologies were evaluated through a general assesment of the calls, as well as through comparison to the DGV [19], HG002 integrated call-set [22] and Conrad et al. (2010) datasets [21]. This comparison provided two valuable insights not found through the previous analyses. First, the technologies produce significantly different amounts of variants: both linked-read WGS and optical mapping produce more than 10,000 calls on a single individual. In contrast, MP WGS generates less than 1000 calls on a single individual. Given the relatively similar detection rate on the CCRs and the fact that nearly all MP WGS calls are found in DGV, the precision of MP WGS is likely to be high compared to optical mapping and linked-read WGS. Second, it is also clear that each method report variants of different types and sizes and that the reported variant not always reflect the nature of the rearrangement in a given sample. This is particularly observed in the optical mapping results, which do not report any duplications at all, even though optical mapping clearly do detect duplications. Similarly, the Long Ranger SV caller for the linked-read WGS data is the only caller to report variants of “unknown” type.

In aggregate, through this comparison, we found that the four WGS methods produce variants of different sizes and these findings are in accordance to the resolution estimates. Notably, there is only a small overlap between the technologies and we only present two orthogonal methods (Bionano optical mapping and Illumina sequencing). Furthermore, many of the calls presented in DGV [19] or Conrad et al. [21] as structural variation are not validated. The results are nevertheless consistent with the previously shown results: MP WGS provides lower detection rate, but higher precision. PCR-free PE WGS, linked-read WGS, and optical mapping detect similar numbers of variants overall, however there are some biases toward certain variant types (for example, optical mapping reports a large number of insertions). Given the similar numbers of detected variants but different numbers of reported variants, the precision of the technologies are likely to be different: we observed that MP WGS provides the highest precision, PCR-free PE WGS the second most precision, and linked-read WGS or optical mapping the worst precision, depending on how the pipelines are combined.

The present study is limited by the fact that we have not compared any third-generation sequencing technologies, which have shown great promise for detection of structural variation in complex repeat regions [30, 31]. However, we found that short-read WGS combined with optical mapping is a powerful combination for analyzing CCRs. Combined, these two technologies would enable detection and validation of most breakpoints in two experiments, at maximum resolution. Given the current high cost of single molecule sequencing, a combined approach could be the most cost efficient. In this study we were able to detect all breakpoint junctions except one using linked-read WGS. The only missing breakpoint junction was in Case 1, where the DNA was partly degraded and no replacement DNA was available due to the individual being deceased. Taken together, and looking at the linked-read WGS result for Case 2 and Case 3, we are confident that the missing junction would have been found with better quality input DNA. Hence, in the cases reported here the linked-read sequencing identified all rearrangement breakpoints including those located in repetitive regions and is a valid WGS method of choice to detail complex rearrangements that often have breakpoints in repetitive regions. However, before this can be used in a clinical setting more user-friendly analysis software, as well as better reference data for filtering is desirable.

In conclusion, these findings demonstrate how different high throughput genomic methods can add clinically relevant information to conventional molecular analysis methods and enable characterization of the true nature of *de novo* CCRs. Finally, Case 3 demonstrates the need for long-molecule sequencing or complementary optical mapping to short-read sequencing to be able to map the structure of a highly complex rearrangement with breakpoints in repetitive regions.

## Materials and methods

Materials and methods for the cytogenetic studies are presented in S1 Appendix and quality control (QC) data of the WGS methods are presented in S5 Table.

### Ethics statement

Written informed consent was obtained from the legal guardians of all study participants. The local ethical boards in Stockholm, Sweden approved the study (approval numbers 2012/2106-31/4 and KS 02–145, 20020506).

### Cases

Three cases were studied following referral to the Clinical Genetics at Karolinska University Hospital, Stockholm, Sweden, due to clinical symptoms indicating genetic testing. Parental samples from Case 2 and Case 3 were analyzed using karyotyping and FISH, showing that the rearrangement had occurred *de novo* on the paternal allele [15, 16]. Parental samples from Case 1 and Case 3 were sequenced using linked-read sequencing, and the rearrangement in Case 1 was also originating from the paternal allele. Clinical parameters and phenotypes of the included cases are presented in Table 10.

**Table 10:** Clinical parameters of included cases.

Clinical	Case 1	Case 2	Case 3
**Gender**	F	M	F
**Birth (weeks)**	40	33	41
**Weight (g) (SD)**	2600 (-1.6)	1800 (-3)	3040 (-0.7)
**Length (cm) (SD)**	46 (-1.6)	48 (-0.7)	49 (-0.2)
**OFC (cm) (SD)**	33 (-1.2)	N.i.	35 (+/-0)
**ID**	No	Yes	Yes
**Hypotonia**	Yes	No	Yes
**Dysmorphic features**	Long philtrum, thin upper lip, hypertelorism, flat nasal bridge	High forehead, downslanting palpebral fissures, hypertelorism, midface hypoplasia, short neck, micrognathia, bristly hair, long and flat philtrum, small mouth, small and low set posteriorly rotated ears	Flat nasal bridge, pointed chin, deep-set eyes
**Autism**	No	Yes	No
**Developmental delay**	Speech and motor functions	Slightly delayed motor functions	Psychomotor
**Frequent infections**	Upper respiratory tract	No	Upper respiratory tract
**Other**	Malrotation of small intestines, failure to thrive, severe feeding difficulties	Cleft lip and palate, hydrocephalus, bilateral deafness, stiff walking	Infectious asthma, unsteady walking, can only construct two word sentences at age 4 years

### Whole genome sequencing

#### Short-read whole genome sequencing

Genomic DNA from three individuals were sequenced using two separate Illumina WGS protocols, a 30X PCR free protocol, as well as a 3X 2.5 kb insert-size mate-pair protocol, at NGI (National Genomics Infrastructure), Stockholm, Sweden (https://ngisweden.scilifelab.se/). The data was processed using the NGI-piper and structural variants were analyzed using the FindSV pipeline (https://github.com/J35P312/FindSV), a pipeline combining CNVnator V0.3.2 [29] and TIDDIT V2.2.4 [10]. Briefly, CNVnator detects CNVs based on read depth signatures, while TIDDIT detects a wide range of SVs by searching for clusters of discordant pairs and split-reads. Hence these callers are complementary, and together they make use of all SV signatures within the WGS data. The outputs of these two callers were combined into one single Variant Calling Format (VCF) file, which was annotated by variant effect predictor (VEP) 89 [32] and filtered based on the VCF file quality flag. The VCF file was subsequently sorted based on a local structural variant frequency database. The PCR-free PE calls were ranked using a database built from the SweGen cohort [17], while the MP WGS dataset was filtered using an in-house database consisting of 55 samples. Variants identified by TIDDIT [10], CNVnator [29], or any of the other methods were visualized using the Integrative Genome Viewer Version 2.4.10 (IGV: http://software.broadinstitute.org/software/igv/) [33] and the exact position of the breakpoint junctions could then be determined by alignment of the split reads to the Hg19 reference genome using the BLAST-like alignment tool (BLAT: https://genome.ucsc.edu/cgi-bin/hgBlat) [34]. Lastly, the VCF file was converted into an excel file using the CCCTG_SV script (https://github.com/J35P312/Garbage_heap/tree/master/sv). This script also applies a frequency filter, which removes all variants found in the database, and filters small intergenic SV (<10 kb). For the PCR-free PE WGS data, the script was set to remove all calls supported by less than eight supporting pairs and split reads, while the MP WGS calls were not filtered based on supporting pairs.

#### Linked-read whole genome sequencing

All three individuals were sequenced using the 10X Genomics Chromium WGS protocol. Briefly, the input DNA is kept as intact as possible, and the large DNA fragments are commonly referred to as molecules. The molecules are separated into droplets, and a unique barcode sequence is added to each droplet. The molecules are fragmented into ~300 bp fragments that are sequenced on the Illumina sequencer. These ~300 bp fragments are produced in such a way that the first read of each read-pair contains the barcode. Hence, upon sequencing the reads, the barcode is also sequenced, allowing for the linking of reads belonging to the same molecule (https://www.10xgenomics.com/genome/). The library was prepared using the 10X Chromium controller and sequenced using the Illumina Hiseq Xten platform at NGI Stockholm. The resulting WGS data was analyzed using two separate pipelines: the default Long Ranger pipeline V2.1.2 (https://support.10xgenomics.com/genome-exome/software/downloads/latest), as well as a custom *de novo* assembly pipeline based on the output of the Supernova V2.0.0 *de novo* assembler (https://support.10xgenomics.com/de-novo-assembly/software/downloads/latest). The custom *de novo* assembly pipeline includes mapping of the raw Supernova contigs using the bwa mem intra-contig mode, and extraction of split contigs using a python script (version 0.1.0) (https://github.com/J35P312/Assemblatron).

#### Optical mapping

Bionano Genomics (San Diego, CA, USA) produced optical maps by running dual enzymes (BspQI, BssSI) on the Saphyr platform (https://bionanogenomics.com/support-page/saphyr-system). The optical maps were analyzed using Bionano-solve (https://bionanogenomics.com/support-page/bionano-solve). Briefly, the maps were detected using AutoDetect (version 5.0 svn:DM:r837), and assembled using the *de novo* assembly package AssembleMolecules (version 1.0). The resulting consensus maps were aligned to hg19 using the Bionano RefAligner (version 5649). Lastly, the Smaps were converted to VCF using a custom version of the smap2vcf script (https://github.com/J35P312/smap2vcf), and the variants of interest were visualized using Bionano access.

### Evaluation of structural variant calling

All three cases were analyzed using a custom designed 400K aCGH [35] and a 1M medical research exome array provided by Oxford Gene Technologies (OGT) (Begbroke, Oxfordshire, UK) (Catalog no. 020100) with exon-resolution in all known medically relevant genes (https://www.ogt.com/products/971_cytosure_medical_research_exome_array). The resulting CNVs were converted into VCF files using the array2vcf script (https://github.com/J35P312/convert2vcf). The variant calls produced by the WGS technologies or optical mapping were compared to these CNVs using SVDB merge V1.1.2. A CNV found by both aCGH and any other technology was considered to be a true positive, all other CNVs were assumed to be false positives.

The variant calls of Case 2 were compared to the DGV, an integrated SV call-set produced by GIAB (ftp://ftp-trace.ncbi.nlm.nih.gov/giab/ftp/data/AshkenazimTrio/analysis/NIST_SVs_Integration_v0.6/README_SV_v0.6.txt), and the list of CNVs presented in Conrad et al. from 2010 [21]. The DGV and the Conrad et al. datasets were converted into VCF files using the DGV2vcf and Conrad2vcf scripts (https://github.com/J35P312/convert2vcf). The resulting VCF files were then split into one VCF per variant type, and compared to the WGS technologies using SVDB merge V1.2.2, which was run using the compare_conrad and compare_dgv scripts (https://github.com/J35P312/convert2vcf). The integrated GIAB call-set was downloaded from the GIAB FTP (ftp://ftp-trace.ncbi.nlm.nih.gov/giab/ftp/data/AshkenazimTrio/analysis/NIST_SVs_Integration_v0.6/HG002_SVs_Tier1_v0.6.vcf.gz), and filtered for variants detected using PacBio or Complete Genomics data. The filtering was performed by reading the CGcalls and PBcalls entries in the info column of the VCF file, and any variant having non-zero CGcalls or PBcalls value were kept while all other variants were filtered out.

Finally, the four technologies were compared by computing intersect of each pairwise technology-combination, and by calculating the sizes and number of variant calls. These scripts are also made available through (https://github.com/J35P312/convert2vcf). Across all comparisons, two variants were considered similar if their overlap exceed a Jaccard index of 0.4, and if the distance between the breakpoints is less than 100 kb. Any variants not meeting these criteria were considered dis-similar. Throughout these comparisons, only high quality calls were considered: a call was considered to be of high quality if the VCF filter flag was set to PASS.

### Breakpoint junction PCR

Primers flanking all junctions except junction 1 and junction 2 in Case 3 were designed approximately 500 base pairs away from the estimated breakpoints. Same primers were subsequently used for sequencing using the Sanger method. Primer sequences are available on request. Breakpoint PCR was performed by standard methods using Phusion High-Fidelity DNA Polymerase (ThermoFisher Scientific, Waltham, MA, USA). Sequences obtained were aligned using BLAT (UCSC Genome Browser) [34] and visualized in CodonCode Aligner (CodonCode Corp., Dedham, MA, USA).

## Supporting information

S1 Fig Results from cytogenetic analysis of Case 1. Karyotyping revealed a large deletion on chromosome 15 and prompted further analysis with fluorescence *in situ* hybridization (FISH), which revealed that the derivative chromosome 15 was part of a complex translocation involving chromosomes 2, 8 and 15 with deletions on chromosome 2 and 8. Spectral karyotyping (SKY) visualized the rearrangement and confirmed the involvement of chromosomes 2, 8 and 15.
(PDF)

S2 Fig Breakpoint junction sequences of all sequences that could be mapped to the nucleotide level. Junction 3 and 4 in Case 1 had two indels and a single nucleotide insertion, respectively, while remaining two junctions were simple. Junction 7 in Case 3 had a non-templated insertion, remaining junctions in Case 3 were simple. All breakpoint junctions in Case 2 were simple. Little (2–6 nucleotides) to no microhomology was observed in all junctions. Lower case letters indicate deletions, and purple indicates microhomology.
(PDF)

S1 Table Ranking of structural variants called by FindSV from short-read mate-pair (MP) and PCR-free paired-end (PE) whole genome sequencing (WGS) data. In order to simulate a scenario where conventional cytogenetic methods had not previously detected the chromosomal rearrangements, we performed structural variant calling, filtering and ranking based on supporting reads and size. Calls supporting the rearrangements were ranked high in all cases except for the paired-end (PE) WGS data for Case 3, which reported 34 interchromosomal variants previous to the intrachromosomal rearrangement on chromosome 1.
(XLS)

S2 Table Sensitivity assessment for all technologies. Sensitivity for each technology was assessed by comparing the generated structural variant calls with copy number variant (CNV) calls from array comparative genomic hybridization (aCGH). A deletion or duplication called by aCGH and any of the technologies was considered a true call.
(XLS)

S3 Table Comparison of different types and sizes of structural variants called by the different technologies. Comparison of the different types and sizes of structural variants called by the different WGS technologies, as well as a calculation of the amount of calls overlapping between the technologies.
(XLS)

S4 Table Size distributions of generated calls for each dataset. (XLS)

S5 Table Quality control (QC) statistics for each method and individual. (XLS)

S1 Appendix Materials and Methods and Results for the cytogenetic studies using karyotyping, fluorescence in situ hybridization (FISH) and array comparative genomic hybridization (aCGH). (DOCX)

S1 Dataset Optical maps supporting all breakpoint junctions identified using the optical mapping technology. (ZIP)
