# Supplementary figures and images for: Comprehensive structural variation genome map of individuals carrying complex chromosomal rearrangements
Source: PLoS Genet. 2019 Feb 8;15(2):e1007858. doi: 10.1371/journal.pgen.1007858 (PMC6368290; doi:10.1371/journal.pgen.1007858)

Case 1 - Karyotype

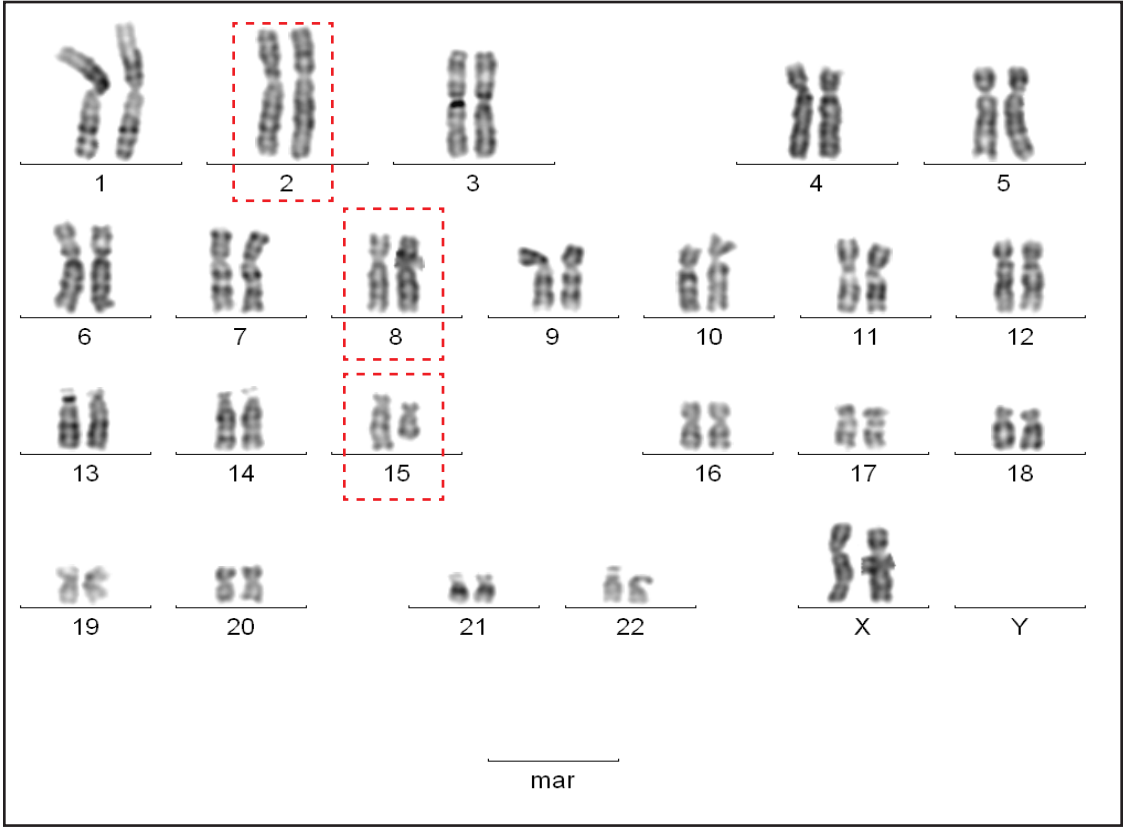

Case 1 - FISH

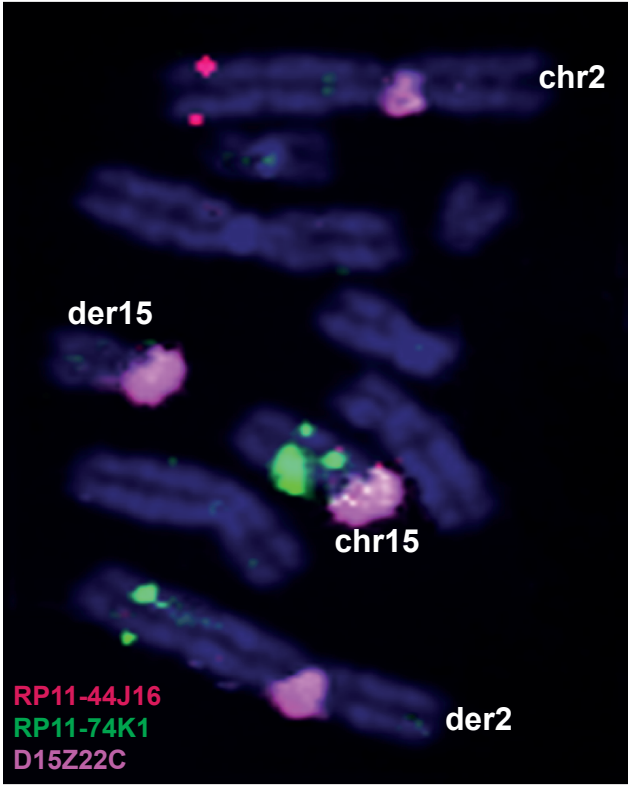

Case 1 - SKY

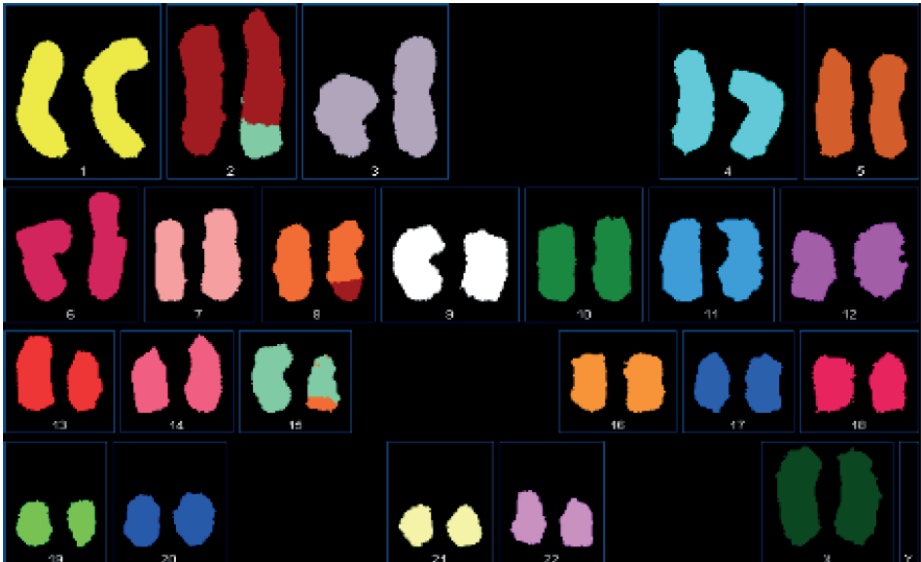

Supplement: S1 Fig — Karyotyping revealed a large deletion on chromosome 15 and prompted further analysis with fluorescence in situ hybridization (FISH), which revealed that the derivative chromosome 15 was part of a complex translocation involving chromosomes 2, 8 and 15 with deletions on chromosome 2 and 8. Spectral karyotyping (SKY) visualized the rearrangement and confirmed the involvement of chromosomes 2, 8 and 15. (PDF) [file pgen.1007858.s001.pdf]
