# Supplementary material for: Comprehensive structural variation genome map of individuals carrying complex chromosomal rearrangements
Source: PLoS Genet. 2019 Feb 8;15(2):e1007858. doi: 10.1371/journal.pgen.1007858 (PMC6368290; doi:10.1371/journal.pgen.1007858)

Supplementary Figure S2

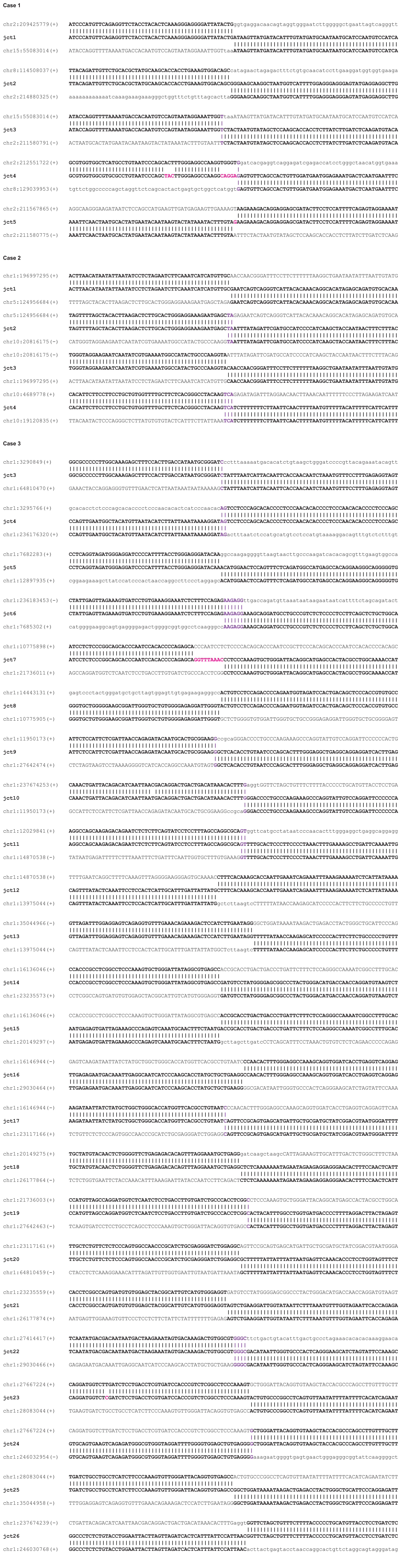

Supplement: S2 Fig — Junction 3 and 4 in Case 1 had two indels and a single nucleotide insertion, respectively, while remaining two junctions were simple. Junction 7 in Case 3 had a non-templated insertion, remaining junctions in Case 3 were simple. All breakpoint junctions in Case 2 were simple. Little (2–6 nucleotides) to no microhomology was observed in all junctions. Lower case letters indicate deletions, and purple indicates microhomology. (PDF) [file pgen.1007858.s002.pdf]
