# Supplementary material for: Comprehensive structural variation genome map of individuals carrying complex chromosomal rearrangements
Source: PLoS Genet. 2019 Feb 8;15(2):e1007858. doi: 10.1371/journal.pgen.1007858 (PMC6368290; doi:10.1371/journal.pgen.1007858)
Supplement: S1 Appendix — (DOCX) [file pgen.1007858.s008.docx]

**Supplementary Methods**

**Cytogenetic methods**

**Karyotyping**

Metaphase slides were prepared from peripheral blood cultures according to standard procedures. Chromosome analysis was performed according to routine procedures using GTG-banding techniques, with a resolution of approximately 550 bands per haploid genome.

**FISH-mapping**

Human genomic BACs/PACs mapped to human chromosome 1, 2, 5, 8, 10 and 15 were chosen based on their mapped position (www.ensembl.org) and ordered from The Wellcome Trust Sanger Institute and delivered as bacterial LB agar stab cultures. The clones were isolated and prepared according to standard protocols. The probes were labelled with FITC and SpectrumOrange by nick translation or random priming, and hybridized and visualized as previously described [1-3]. A minimum of 10 metaphases per hybridization was analyzed.

**Array-CGH**

DNA was extracted from peripheral blood using standard protocols. Two array designs were used: one custom designed 2x400K array for the first copy number variant (CNV) screening, and one 1x1M medical research exome array provided by Oxford Gene technology (OGT) (Begbroke, Oxfordshire, UK) (Catalog no. 020100) for the method comparison. We used eArray (https://earray.chem.agilent.com/earray/) to customize the array design for the 2x400K design, the Agilent online web tool for array design, and array slides for both designs were subsequently ordered from OGT. The procedures for the array experiments were as described previously [4]. CNVs were called if four probes in a row were aberrant and thresholds for calling duplications were log2 Cy3/Cy5 ratio of +0.3 for duplications and -0.6 for deletions. All aberrations called by the software were manually inspected and classified regarding to the clinical phenotypes according to the American College of Medical Genetics (ACMG) guidelines [5]. According to the manufacturer (OGT), optimal DLR spread value for analysis and detection of small variants is <0.20, with thresholds given as follows: <0.20 Excellent, <0.30 Good, >0.30 Poor (Evaluate).

**Supplementary Results**

All coordinates are in Hg19/GRCh37.

**Case 1 cytogenetic studies**

Regular karyotyping of Case 1 revealed an apparently balanced complex translocation between the long arm of chromosome 2, the long arm of chromosome 8 and the long arm of chromosome 15. The karyotypes of both parents were normal. Array-CGH data was obtained from the 2x400K custom design and the 1M medical exome array design with derivative log ratio (DLR) spread values of 0.21 and 0.27, respectively. Array-CGH confirmed both deletions on chromosome 2 (2.1 Mb, 2q34(209,491,014-211,575,180)x1 and 2.3 Mb, 2q34(212,563,529-214,883,765)x1) and the deletion on chromosome 8 (14.5 Mb, 8q23.3q24.21(114,503,675-129,066,509)x1). Numbers of all variants that were detected including polymorphic variants are presented in Table 6. Subsequent high resolution FISH-mapping of the rearrangement confirmed the deletions. The same FISH-clones were not deleted in hybridizations of samples from the parents. On chromosome 2 the proximal breakpoint was localized between RP11-299P7 and RP11-101B5 (deleted) and the distal breakpoint between the clone RP11-44J16 (deleted) and RP11-319N19 (signal at 8q23), thus limiting the deletion to 5.0 Mb, localized 209.4-214.4 Mb from the 2p-telomere. On chromosome 8 the proximal breakpoint was localized between RP11-252H24 and RP11-267L5 (deleted) and the distal breakpoint between the clone RP11-125A17 (deleted) and RP11-294P07 (signal at 15q21), thus limiting the deletion to 14.1 Mb, localized 115.0-129.1 Mb from the 8p-telomere. The breakpoint on chromosome 15 was located between the clone RP11-215J7 and RP11-455L19 (signal at 2q34).

**Case 2 cytogenetic studies**

Chromosome analysis of Case 2 revealed a *de novo* apparently balanced complex chromosome rearrangement involving chromosomes 1, 5 and 10: 46,XY,t(1;10;5)(q32;p12;q31), and subsequent FISH mapping and BAC array revealed a deletion of 14.5 Mb within the translocated segment of chromosome 10, as reported previously [2]. Array-CGH with a custom 2x400K array design and 1M medical exome array design yielded data with DLR spread values of 0.18 and 0.26, respectively, and confirmed the deletion on chromosome 10 (14.4 Mb, 10p15.1p12.31(4,701,247-19,158,598)x1). Numbers of all variants that were detected including polymorphic variants are presented in Table 6. Parental samples were analyzed using FISH and showed that the rearrangement had occurred on the paternal allele [2].

**Case 3 cytogenetic studies**

Karyotyping of Case 3 showed an unusual banding pattern on chromosome 1. Subsequent FISH analysis showed a split signal at 1p36.32 and 1p31.3. Over 130 different BAC-clones were used and revealed an extremely complex *de novo* rearrangement of chromosome 1 with 14 breakpoints, involving both p- and q-arm. A small deletion of approximately 0.5 Mb was identified on 1p36.2 using BAC array and confirmed with FISH mapping [1]. Array-CGH with custom 2x400K array design and 1M medical exome array design yielded data with DLR spread values of 0.15 and 0.22, respectively, and confirmed the deletion on 1p (840kb, 1p36.22p36.21(12,026,731-12,866,505)x1).

**References**

1. Lindstrand A, Malmgren H, Sahlen S, Xin H, Schoumans J, Blennow E. Molecular cytogenetic characterization of a constitutional, highly complex intrachromosomal rearrangement of chromosome 1, with 14 breakpoints and a 0.5 Mb submicroscopic deletion. American journal of medical genetics Part A. 2008;146A(24):3217-22.

2. Lindstrand A, Malmgren H, Verri A, Benetti E, Eriksson M, Nordgren A, et al. Molecular and clinical characterization of patients with overlapping 10p deletions. American journal of medical genetics Part A. 2010;152A(5):1233-43.

3. Malmgren H, Sahlen S, Wide K, Lundvall M, Blennow E. Distal 3p deletion syndrome: detailed molecular cytogenetic and clinical characterization of three small distal deletions and review. American journal of medical genetics Part A. 2007;143A(18):2143-9.

4. Pettersson M, Viljakainen H, Loid P, Mustila T, Pekkinen M, Armenio M, et al. Copy Number Variants Are Enriched in Individuals With Early-Onset Obesity and Highlight Novel Pathogenic Pathways. J Clin Endocrinol Metab. 2017;102(8):3029-39.

5. Richards S, Aziz N, Bale S, Bick D, Das S, Gastier-Foster J, et al. Standards and guidelines for the interpretation of sequence variants: a joint consensus recommendation of the American College of Medical Genetics and Genomics and the Association for Molecular Pathology. Genet Med. 2015;17(5):405-24.
